# Role of nerve ultrasound and shear wave elastography in stratified diagnosis of diabetic peripheral neuropathy

**DOI:** 10.1097/MD.0000000000045492

**Published:** 2025-11-07

**Authors:** Zhen-han Lai, Bo-yu She, Meng-lu Song, Jia-ying Wang, Hua-qiang Zheng, Kun-bin Wu, Wen-ting Jiang, Guo-rong Lyu

**Affiliations:** a Department of Ultrasound, Zhangzhou Affiliated Hospital of Fujian Medical University, Zhangzhou, Fujian, China; b The Third Clinical Medical College, Fujian Medical University, Fuzhou, Fujian, China; c Department of Ultrasound, Second Affiliated Hospital of Fujian Medical University, Quanzhou, Fujian, China.

**Keywords:** cross-sectional area, diabetic complications, diabetic peripheral neuropathy, nerve ultrasound, shear wave elastography

## Abstract

The aim of this study is to ascertain the role of high-resolution nerve ultrasound (NUS) and shear wave elastography (SWE) in assessing the stratified diagnosis of diabetic peripheral neuropathy (DPN). To assess the potential diagnostic worth of NUS and SWE, across different levels of DPN. To determine whether the integration of NUS with SWE can enhance diagnostic accuracy. This retrospective study enrolled a total of 121 patients diagnosed with type 2 diabetes mellitus from September 2022 to May 2024, including 91 patients with DPN. All participants were categorized into 4 groups: group A (subclinical-DPN), group B (suspected-DPN), group C (clinically diagnosed-DPN), group D (confirmed-DPN), with a control group of type 2 diabetes mellitus patients without DPN. NUS and SWE examinations were performed to generate the receiver operating characteristics (ROC) curves of different diagnostic methods, determine the diagnostic threshold and compare the difference in diagnostic efficacy. Significant differences were observed in tibial nerve characteristics between DPN patients and controls. The DPN group had a larger cross-sectional area (CSA; 24.78 ± 4.75 mm² vs 22.40 ± 3.19 mm², *P* < .05) and higher mean elasticity modulus (*E*_mean_; 54.46 ± 16.76 kPa vs 34.37 ± 9.37 kPa, *P* < .05). ROC curve analysis was performed to evaluate the diagnostic performance of CSA, *E*_mean_, and combined model (*E*_mean_ + CSA) in detecting DPN. The area under the ROC curve of *E*_mean_ was significantly higher than NUS (*z* = −4.032, *P* < .001). The combined model showed no significant improvement over SWE alone (*z* = −1.486, *P* = .137). Stratified analysis revealed that CSA measurements are more reliable in advanced disease stages; *E*_mean_ demonstrated significantly superior diagnostic efficacy compared to CSA for all disease stages. The combination of *E*_mean_ and CSA showed more pronounced improvements in all disease stages compared to CSA alone, while only showing marginal improvement trends in subclinical and confirmed-DPN, without reaching statistical significance compared to *E*_mean_. This study establishes an evidence-based, stage-specific framework for the diagnosis of DPN. The combined NUS + SWE approach offers unparalleled advantages for early detection, whereas SWE alone may suffice in advanced stages.

## 1. Introduction

The incidence of diabetes mellitus (DM) has witnessed a steady rise in recent years, propelled by the rapid growth of the global economy, improvements in living standards, and changes in lifestyle habits and dietary patterns. According to the 2021 International Diabetes Federation data, the global prevalence of diabetes among adults is estimated at 537 million, with projections indicating a surge to 783 million by 2045.^[1]^ Diabetic peripheral neuropathy (DPN), the most prevalent chronic complication of DM, arises from prolonged hyperglycemia-driven microvascular damage, oxidative stress, and mitochondrial dysfunction, leading to axonal degeneration and demyelination. Clinically, DPN typically presents with distal symmetric pain, paresthesia, and sensory loss, which may progress to foot ulcers and Charcot arthropathy in advanced stages. Thus, early diagnosis and intervention are crucial, as irreversible nerve damage often occurs when symptoms manifest.

Currently, clinical diagnosis of DPN relies on symptoms and signs, but standardized criteria are lacking. However, the detection rate of DPN varies significantly across different studies due to the absence of standardized diagnostic criteria.^[2]^ Nerve conduction study, considered one of the gold standard techniques for diagnosing DPN^[3]^ is limited in its clinical application due to its insensitivity to small nerve fibers and unmyelinated nerve fibers, extended duration, high cost, and patient discomfort during examination.^[4]^ Crucially, they are prone to missing diagnoses in patients with advanced DPN. Additionally, subclinical patients may lack obvious symptoms and signs.^[5]^ Thus, noninvasive methods like high-resolution nerve ultrasound (NUS) and shear wave elastography (SWE) are urgently needed for early screening.

With the continuous development of modern medical ultrasound imaging equipment, high-resolution ultrasound along with various imaging modalities has been used to improve early DPN diagnosis due to its ability to clearly visualize nerve structures.^[6–8]^ High-resolution NUS has become an indispensable method for the early screening and diagnosis of DPN due to its superficial peripheral nerve position, and its noninvasive nature, convenience, and affordability. As a diagnostic tool, it has been proved to be practical, widely available, reproducible, with no relevant contraindications and costeffective.^[9]^ At the pathophysiological level, the neural structural changes caused by DPN provide a theoretical basis for imaging diagnosis. The current research suggests that chronic hyperglycemia induces a range of metabolic events in DPN, leading to the accumulation of sorbitol and fructose within peripheral nerves, causing increased osmotic pressure, cellular swelling, and an expansion in the volume of nerve fibers.^[10]^ Another study suggested that nerve bundle edema in patients with DPN could increase intraneural pressure, resulting in increased nerve stiffness.^[11]^ These pathological changes collectively lead to neurofibrosis and connective tissue proliferation, which morphologically manifest as thickened nerves and biomechanically as increased nerve hardness. This provides scientific basis for applying NUS to assess neural morphological changes and SWE to measure tissue elasticity. NUS offers vital details, including neural cross-sectional area (CSA), echogenicity, and internal anatomical features, which enable the assessment of various degrees of DPN. Previous studies^[12,13]^ have reported the potential role of NUS in diagnosing DPN based on the enlarged maximum thickness and CSA of the tibial nerve. Additionally, several scholars have conducted research.^[14]^ On the ultrasonographic alterations of the cervical vagus nerve among individuals diagnosed with DPN, revealing that high-resolution NUS shows thickening of the cervical vagus nerve in patients with DPN, which is a potential diagnostic feature of diabeticneuropathy. However, NUS cannot directly reflect changes in nerve stiffness. Shear wave elastic imaging (SWE), an increasingly prevalent novel measurement method in ultrasonic imaging, enables direct and quantitative assessment of nerve stiffness.^[15,16]^ A systematic review and meta-analysis reported that SWE might improve the specificity and sensitivity of ultrasound examination in the evaluation of DPN.^[17]^ The combined utilization of NUS and SWE techniques enables the assessment of morphological and biomechanical alterations in nerves, serving as a valuable adjunctive tool for diagnosing DPN and offering significant research prospects. Although diagnostic criteria for DPN using NUS and SWE have been discussed in the literature,^[8,18]^ there few reports stratified studies on severity.^[19]^

Based on the aforementioned background, the purpose of this study is to systematically evaluate the diagnostic value of high-resolution NUS and SWE for DPN through retrospective analysis, and to focus on the following scientific questions: Are there differences in the neurosonographic features of patients with DPN at different stages of development; What are the characteristics of the diagnostic efficacy of NUS and SWE at different stages of DPN; can the combined application of the 2 technologies significantly improve diagnostic efficiency? It is hypothesized that neuromorphological and elastic property changes exhibit phased characteristics with DPN progression; NUS and SWE have complementary diagnostic values at different stages; the combined application of the 2 technologies can significantly improve the diagnostic accuracy.

## 2. Materials and methods

### 2.1. Subjects

This retrospective study enrolled 121 type 2 diabetes mellitus (T2DM) patients (242 nerves) diagnosed with diabetes who were admitted to our endocrinology department between September 2022 and May 2024. Among them, 91 patients (182 nerves) were identified with DPN, while the remaining 30 patients (60 nerves) served as the control group without diabetic peripheral neuropathy (NDPN). Following the diagnostic stratification criteria for diabetic neuropathy established by the Diabetes Branch of the Chinese Medical Association, DPN subgroups were defined per Chinese Medical Association guidelines.^[20]^ DPN patients were further classified into 4 subgroups: subclinical-DPN group consisted of 26 cases (52 nerves), suspected-DPN group consisted of 19 cases (38 nerves), clinical-DPN group included 20 cases (40 nerves), and confirmed-DPN group comprised 26 cases (52 nerves).

The criteria for inclusion were as follows: all patients met the Chinese Guidelines for the Prevention and Treatment of Diabetes Mellitus (2024 Edition) diagnostic criteria for T2DM.^[21]^ The criteria for exclusion were as follows: type 1 DM, multiple neuropathy or secondary neuropathy caused by other reasons, the use of chemotherapeutic drugs may cause neurotoxicity and renal insufficiency may cause nerve damage caused by metabolic poisons, the onset time of neuropathy was earlier than the diagnosis time of diabetes, patients who did not undergo nerve conduction velocity examination and foot vibration perception threshold examination or whose examination results were incomplete.

This study was approved by the Ethics Committee of Zhangzhou Affiliated Hospital of Fujian Medical University (2022KY299). At the time of the examination, all patients gave their written consent for the scientific collection and evaluation of image data.

### 2.2. Electrodiagnostic examinations

The electrodiagnostic examinations were conducted according to the protocol proposed by O'Bryan et al.^[22]^ This study employed the KEYPOINT 9033A07 4-direction EMG induction potentiometer manufactured by Denmark.

### 2.3. Nerve ultrasound (NUS) examinations

**Figure 1.**
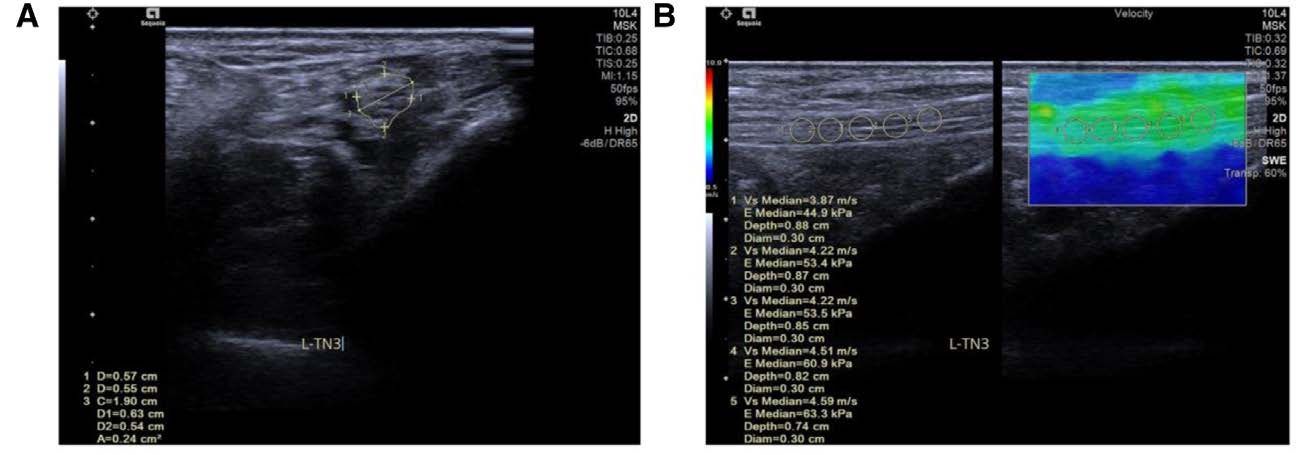
Ultrasound diagnosis. (A) The cross section of the NUS reveals showed tibial nerve CSA was measured to be 0.24 cm^2^; (B) the longitudinal section of NUS showed the perineurium of tibial nerve of the inner ankle is slightly blurred, ROI: 3 mm × 3 mm; measurements taken at medial malleolus. TN: tibial nerve. CSA = cross-sectional area, NUS = nerve ultrasound, ROI = regions of interest.

Nerve sonography was performed on all subjects by a ultrasonographer with more than 10 years of experience in peripheral nerve ultrasonography. The subjects’ clinical condition was concealed from the examiner. Ultrasound imaging was conducted using a 12L-5 transducer (5–12 MHz; Acuson S3000; Siemens AG). Participants were instructed to assume a prone position on the examination table with legs slightly separated and feet in an internally rotated position. A generous quantity of coupling agent was applied to the skin, and the transducer was placed transversely posterior to the medial malleolus, where the posterior tibial artery and vein along with the tibial nerve can be visualized posterior to the flexor digitorum longus tendon. Subsequently, both proximal and distal tracking of the tibial nerve can be performed from this reference point. During transverse scanning, the CSA of the tibial nerve should be measured at its location within the medial retromalleolar groove (Fig. 1A). The CSA was measured 3 times for each participant, and each examination was repeated 3 times by the same ultrasound physicians to enhance reproducibility. The mean value was chosen for subsequent analysis.

### 2.4. Shear wave elastography (SWE) examinations

An image was generated using a 5 to 12 MHz transducer combined with a 12L-5 transducer and SWE. The ultrasound physician positioned the transducer over the tibial nerve at the medial malleolus. Subsequently, the transducer was rotated by 90 degrees and moved along the longitudinal axis of the nerve for scanning (Fig. 1B). The shear wave’s quality measure was evaluated using the quality control model. In the image, a green region is marked as the high-quality area, while other areas are considered of poor quality. Regions of interest (ROI) were designated within the high-quality zone. The energy mode was activated in this area to measure the modulus of elasticity of the tibial nerve. The lower limit for the ROI range was set at 3 mm^2^ (obtained by multiplying 3 mm by 3 mm). ROI measurements were performed 5 times at medial malleolus (Fig. 1B). The maximum as well as the minimum values were removed and the remaining data were averaged. The double-blind method was employed to measure all parameters, and the mean elasticity modulus (*E*_mean_) was calculated as a representative result.

### 2.5. Statistical analysis

All data analyses were performed by SPSS software (IBM Corp. Released in 2015. IBM SPSS Statistics for Windows, Version 23.0. Armonk: IBM Corp.), statistical analyses were supervised by a professional biostatistician. The Anderson-Darling normality test was used for evaluation of normal distribution. The continuous variables were presented as the mean ± standard deviation, while categorical variables were shown as numbers. For comparing categorical variables, either the Chi-squared test or Fisher’s exact test was utilized. Comparisons CSA and *E*_mean_ of the tibial nerve between DPN group and control group were made for continuous variables using Student *t* test. Receiver operating characteristic (ROC) curves were plotted to identify the optimal cutoff values of CSA and *E*_mean_ for diagnosing DPN. Sensitivity, specificity, and the area under the ROC curve (AUC) were calculated. Additionally, 1-way ANOVA was applied to assess differences in continuous variables among groups stratified by DPN severity. The DeLong test compares the disparities in AUC. Statistical significance was established at a *P*-value of <.05.

## 3. Results

### 3.1. Comparison of clinical and ultrasonographic characteristics between DPN and NDPN groups

Based on the inclusion and exclusion criteria, a total of 121 cases (91 DPN patients and 30 controls) with T2DM were included in the study, comprising 242 nerves (182 nerves in the DPN group and 60 nerves in the NDPN group). There were no significant differences in age, gender, body mass index (BMI) between the 2 groups (*P* > .05). The group with DPN exhibited a significantly higher CSA value when compared to the control NDPN group (24.78 ± 4.75) mm^2^ vs (22.40 ± 3.19) mm^2^ (*P* < .01), while *E*_mean_ value in the group with DPN was also significantly higher compared to the control NDPN group (54.46 ± 16.76) kPa vs (34.37 ± 9.37) kPa (*P* < .01; Table 1).

**Table 1:** Comparison of clinical and ultrasonographic characteristics between DPN and NDPN groups.

Characteristic	DPN (n = 182)	NDPN (n = 60)	*P*
Age (yr)	51.56 ± 11.35	49.42 ± 10.11	.194
Gender (M/F)	123/59	40/20	.896
BMI (kg/m^2^)	23.59 ± 3.63	24.57 ± 3.23	.064
CSA (mm^2^)	24.78 ± 4.75	22.40 ± 3.19	<.001
*E*_mean_ (kPa)	54.46 ± 16.76	34.37 ± 9.37	<.001

BMI = body mass index, CSA = cross-sectional area, DPN = diabetic peripheral neuropathy, *E*_mean_ = mean elastic modulus, M/F = male/female ratio, NDPN = without diabetic peripheral neuropathy.

### 3.2. Diagnostic performance of NUS, SWE, and combined NUS + SWE models for DPN

**Figure 2.**
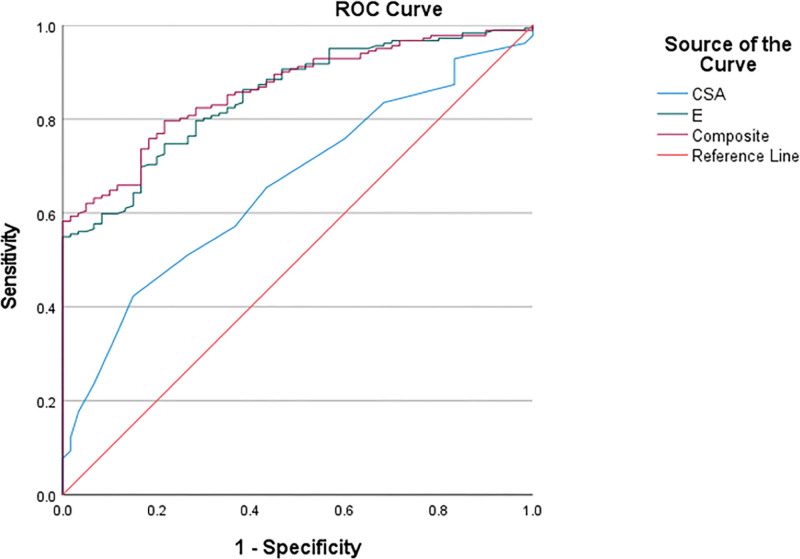
ROC curve of CSA (AUC = 0.658), *E*_mean_ (AUC = 0.849), and *E*_mean_ + CSA (AUC = 0.862) values in diagnosing DPN. AUC = area under the ROC curve, CSA = cross-sectional area, DPN = diabetic peripheral neuropathy, *E*_mean_ = mean elasticity modulus, ROC = receiver operating characteristic.

ROC curve analysis was performed to evaluate the diagnostic performance of CSA, *E*_mean_, and joint diagnostic model (*E*_mean_ + CSA) in detecting DPN (Fig. 2). The AUC for CSA alone was .658, indicating moderate discriminative ability. In contrast, *E*_mean_ demonstrated a significantly higher diagnostic accuracy with an AUC of .849. *E*_mean_ + CSA further improved diagnostic performance, achieving the highest AUC of .862. Statistical analysis using DeLong’s test (Table 2) revealed that *E*_mean_ demonstrated significantly superior diagnostic efficacy compared to CSA (*z* = −4.032, *P* < .001). *E*_mean_ + CSA shows even more significant improvement. Compared to CSA alone, its advantages are more pronounced (*z* = −4.988, *P* < .001). Furthermore, the analysis revealed significant differences in diagnostic performance among various models across DPN stages. For the overall DPN cohort, *E*_mean_ demonstrated significantly superior diagnostic efficacy compared to CSA (*z* = −4.032, *P* < .001). This advantage was maintained across all disease stages: subclinical (*z* = −2.769, *P* = .006), suspected (*z* = −2.795, *P* = .005), clinical (*z* = −3.698, *P* < .001), and confirmed-DPN (*z* = −2.996, *P* = .003).

**Table 2:** Diagnostic performance of NUS, SWE, and combined NUS + SWE models for DPN.

Model	Parameter	Cutoff value	Sensitivity (%)	Specificity (%)	Accuracy (%)	AUC (95% CI)	AUC comparison (*z*/*P*-value)
NUS	CSA	25.50	42.3	85.0	74.0	0.658 (0.584–0.732)	CSA vs *E*_mean_: *z* = −4.032, *P* ≤ .001
SWE	*E*_mean_	51.68	54.9	100.0	80.2	0.849 (0.800–0.898)	*E*_mean_ vs CSA + *E*_mean_: *z* = −1.486, *P* = .137
NUS + SWE	CSA + *E*_mean_		58.2	100.0	81.0	0.862 (0.817–0.908)	CSA + *E*_mean_ vs CSA: *z* = −4.988, *P* ≤ .001

AUC = area under the ROC curve, CI = confidence interval, CSA = cross-sectional area (mm²), DPN = diabetic peripheral neuropathy, *E*_mean_ = mean elastic modulus (kPa), NUS = nerve ultrasound, SWE = shear wave elastography.

*E*_mean_ + CSA showed more pronounced improvements. Compared to CSA alone, its advantage was most prominent in overall DPN (*z* = −4.988, *P* < .001), with statistical significance observed in subclinical (*z* = −3.281, *P* = .001), suspected (*z* = −3.516, *P* < .001), clinical (*z* = −4.630, *P* < .001), and confirmed-DPN (*z* = −3.890, *P* < .001). Notably, comparison between *E*_mean_ + CSA and *E*_mean_ alone showed only marginal improvement trends in subclinical (*z* = −1.014, *P* = .311) and confirmed-DPN (*z* = −1.538, *P* = .124), without reaching statistical significance.

### 3.3. Comparison of clinical and ultrasonographic characteristics between 4 DPN diagnostic classification

Further analysis categorized DPN patients into subclinical-, suspected-, clinical-, and confirmed-DPN subgroups (Table 3). There were no significant differences in age and gender distribution among the subgroups (*P* > .05). BMI exhibited notable variation (*P* < .001), with the suspected-DPN group having the highest BMI (26.20 ± 4.63 kg/m²). Both tibial nerve CSA and *E*_mean_ showed statistically significant differences across subgroups (both *P* < .001). CSA values progressively increased from subclinical-DPN (22.62 ± 4.43 mm²) to confirmed-DPN (28.52 ± 4.64 mm²), with the most pronounced difference observed between confirmed-DPN and both subclinical-DPN (*P* < .001) and clinical-DPN (*P* < .001). *E*_mean_ values demonstrated a similar trend, rising from 45.12 ± 14.01 kPa in subclinical-DPN to 70.37 ± 13.83 kPa in confirmed-DPN. Pairwise comparisons revealed significant elevations in confirmed-DPN versus subclinical-DPN (*P* < .001) and clinical-DPN (*P* < .001). Notably, no significant differences in CSA or *E*_mean_ were found between suspected-DPN and clinical-DPN groups (*P* = .734 and *P* = .972, respectively).

**Table 3:** Comparison of clinical and ultrasonographic characteristics between 4 DPN diagnostic classification.

Clinical classification	Subclinical-DPN (n = 52)	Suspected-DPN (n = 38)	Clinical-DPN (n = 40)	Confirmed-DPN (n = 52)	Overall *P*-value	Pairwise *P*-value*
Age (yr, *x̄* ± s)	51.44 ± 13.75	55.63 ± 12.15	53.13 ± 7.52	54.81 ± 8.88	.511	a = .142, b = .412, c = .328
Gender (male/female)	32/20	28/10	24/16	39/13	.428	a = .143, b = .204, c = .134
BMI (kg/m^2^, *x̄* ± s)	23.29 ± 2.82	26.20 ± 4.63	23.55 ± 3.35	22.03 ± 2.67	<.001	a = .022, b = .005, c = .018
CSA (mm^2^, *x̄* ± s)	22.62 ± 4.43	23.87 ± 3.69	23.60 ± 3.26	28.52 ± 4.64	<.001	*a* < .001, b = .734, c < .001
*E*_mean_ (kPa, *x̄* ± s)	45.12 ± 14.01	50.04 ± 13.83	50.14 ± 11.16	70.37 ± 13.83	<.001	a < .001, b = .972, c < .001

BMI = body mass index, CSA = cross-sectional area, DPN = diabetic median neuropathy, *E*_mean_ = mean value of the modulus of elasticity.

* Pairwise comparison key: a: subclinical vs confirmed-DPN; b: suspected vs clinical-DPN; c: clinical vs confirmed-DPN.

### 3.4. Diagnostic performance of NUS, SWE, and combined NUS + SWE models for DPN in subgroups

**Figure 3.**
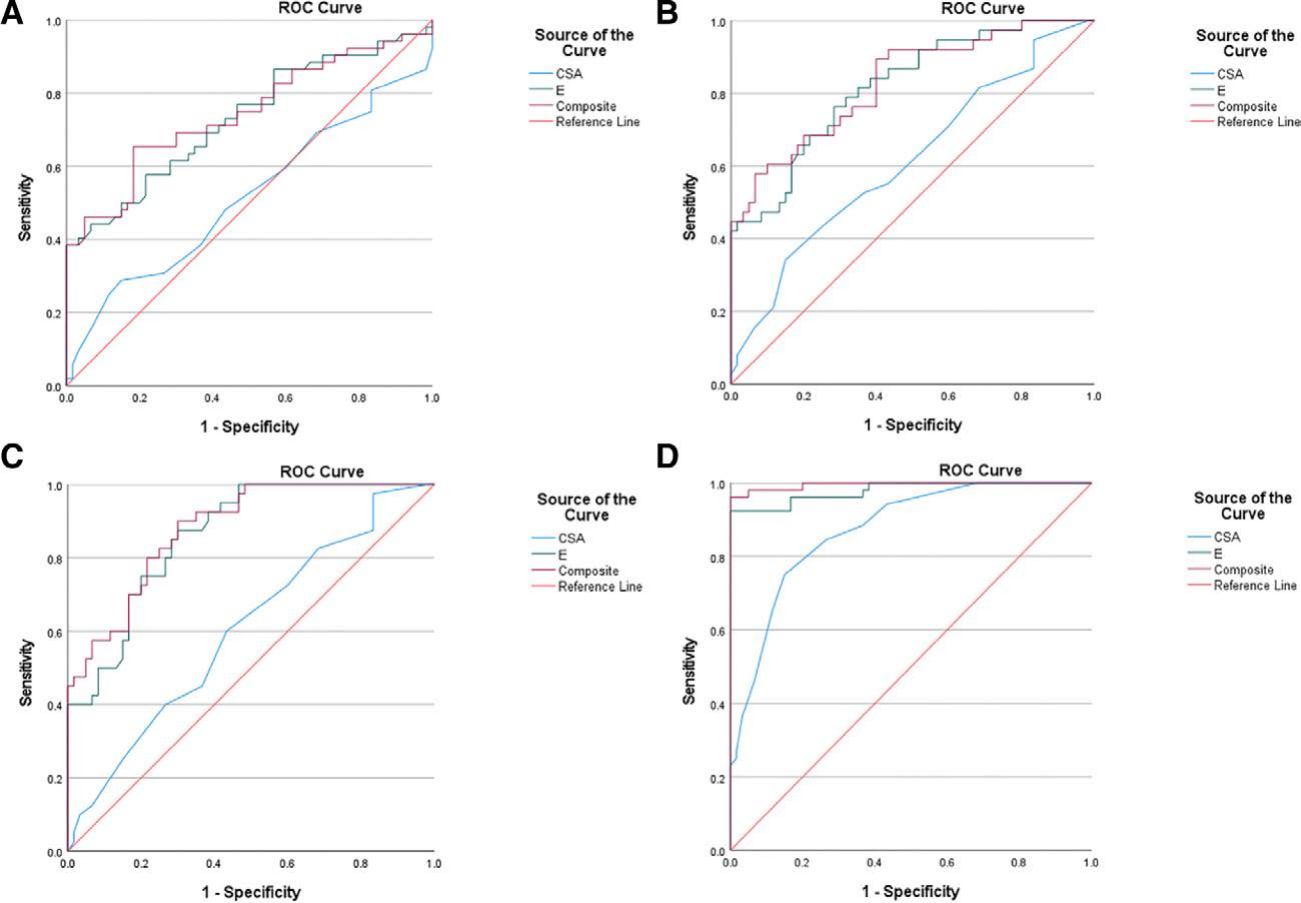
(A) ROC curve for CSA (AUC = 0.515), *E*_mean_ (AUC = 0.730), and *E*_mean_ + CSA (AUC = 0.744) values in diagnosing subclinical-DPN; (B) ROC curve for CSA (AUC = 0.614), *E*_mean_ (AUC = 0.819), and *E*_mean_ + CSA (AUC = 0.830) values in diagnosing suspected-DPN; (C) ROC curve for CSA (AUC = 0.604), *E*_mean_ (AUC = 0.862), and *E*_mean_ + CSA (AUC = 0.880) values in diagnosing suspected or clinical-DPN; (D) ROC curve for CSA (AUC = 0.874), *E*_mean_ (AUC = 0.979), and *E*_mean_ + CSA (AUC = 0.995) values in diagnosing suspected or confirmed-DPN. AUC = area under the ROC curve, CSA = cross-sectional area, DPN = diabetic peripheral neuropathy, *E*_mean_ = mean elasticity modulus, ROC = receiver operating characteristic.

The diagnostic performance of CSA, *E*_mean_, and *E*_mean_ + CSA was systematically evaluated across different stratifications of DPN, including subclinical-, suspected-, clinical-, and confirmed- DPN (Table 4). A ROC curve was further constructed to illustrate and evaluatethe diagnostic performance of NUS, SWE, and combined NUS + SWE models for DPN in subgroups (Fig. 3).

**Table 4:** Diagnostic performance of NUS, SWE, and combined NUS + SWE models for DPN in subgroups.

Parameter		Cutoff value	Sensitivity (%)	Specificity (%)	Accuracy (%)	AUC (95% CI)	AUC comparison (*P*-value)
Subclinical-DPN	CSA	25.50	28.8	75.0	54.5	0.515 (0.405–0.625)	CSA vs *E*_mean_: .006
	*E*_mean_	51.68	38.5	100.0	67.0	0.730 (0.635–0.825)	*E*_mean_ vs CSA + *E*_mean_: .311
	CSA + *E*_mean_		65.4	81.7	74.1	0.744 (0.650–0.838)	CSA + *E*_mean_ vs CSA: .001
Suspected-DPN	CSA	25.50	34.2	85.0	62.2	0.614 (0.498–0.729)	CSA vs *E*_mean_: .005
	*E*_mean_	39.84	76.3	71.7	72.4	0.819 (0.735–0.903)	*E*_mean_ vs CSA + *E*_mean_: .567
	CSA + *E*_mean_		57.9	93.3	74.5	0.830 (0.746–0.913)	CSA + *E*_mean_ vs CSA: <.001
Clinical-DPN	CSA	22.50	60.0	56.7	60.1	0.604 (0.491-0.716)	CSA vs *E*_mean_: <.001
	*E*_mean_	39.56	87.5	70.0	78.0	0.862 (0.794–0.931)	*E*_mean_ vs CSA + *E*_mean_: .238
	CSA + *E*_mean_		90.0	70.0	76.0	0.880 (0.817–0.944)	CSA + *E*_mean_ vs CSA: <.001
Confirmed-DPN	CSA	25.50	75.0	85.0	80.4	0.874 (0.811–0.937)	CSA vs *E*_mean_: .003
	*E*_mean_	51.79	92.3	100.0	95.5	0.979 (0.956–1.000)	*E*_mean_ vs CSA + *E*_mean_: .124
	CSA + *E*_mean_		96.2	100.0	97.3	0.995 (0.987–1.000)	CSA + *E*_mean_ vs CSA: <.001

AUC = area under the ROC curve, CSA = cross-sectional area (mm²), DPN = diabetic peripheral neuropathy, *E*_mean_ = mean elastic modulus (kPa), *E*_mean_+CSA = CSA combined *E*_mean_, NUS = nerve ultrasound, SWE = shear wave elastography.

Table 4 evaluates the diagnostic efficacy of CSA, *E*_mean_, and *E*_mean_ + CSA at different stratifications of DPN. CSA exhibited moderate sensitivity (28.8–75.0%) but consistently high specificity (56.7–85.0%), with accuracy improving from 54.5% in subclinical-DPN to 80.4% in confirmed-DPN. CSA cutoffs ranged from 22.50 mm² (clinical-DPN) to 25.50 mm² (other stages). This suggests that CSA measurements are more reliable in advanced disease stages. *E*_mean_ demonstrated superior performance, with sensitivity ranging from 38.5% (subclinical-DPN) to 92.3% (confirmed-DPN) and specificity between 71.7% and 100%. *E*_mean_ cutoffs showed greater variation (39.56–51.79 kPa), Notably, SWE achieved the highest accuracy (95.5%) in confirmed-DPN, indicating its robustness in late-stage diagnosis. As previously analyzed before stratified diagnosis, the diagnostic efficacy of *E*_mean_ + CSA approach in the overall DPN cohort (AUC = 0.862, 95% CI: 0.817–0.908) was significantly superior to that of single detection methods (CSA: AUC = 0.658; *E*_mean_: AUC = 0.849). This advantage was verified across all stages of DPN, particularly in early diagnosis (Fig. 3), maintaining high specificity (70.0–100%), supporting its application value in early detection without compromising diagnostic certainty. For subclinical-DPN, the combined model’s AUC reached 0.744 (95% CI: 0.650–0.838), showing a significant improvement over single detection methods (CSA: AUC = 0.515; *E*_mean_: AUC = 0.730; all comparisons *P* < .01). The most significant improvement was in sensitivity (65.4% vs 38.5% for SWE alone).

Table 4 compares the diagnostic efficacy of CSA, *E*_mean_, and *E*_mean_ + CSA across different stratifications of DPN. Stage-specific analysis revealed distinct diagnostic patterns: In subclinical-DPN, the combination model showed marked improvement in sensitivity (65.4%) compared to individual modalities (CSA: 28.8%; *E*_mean_: 38.5%), while maintaining reasonable specificity (81.7%). The AUC increased from 0.515 (CSA) and 0.730 (*E*_mean_) to 0.744 for the combined approach. For suspected-DPN, *E*_mean_ alone exhibited good diagnostic performance (AUC = 0.819, sensitivity = 76.3%), with the combination further enhancing specificity from 71.7% to 93.3% at a slight sensitivity cost (57.9%). The optimal *E*_mean_ cutoff decreased to 39.84 kPa in this stage. Clinical-DPN demonstrated *E*_mean_’s strong diagnostic value (AUC = 0.862, sensitivity = 87.5%), with the combination providing modest AUC improvement to 0.880. Notably, CSA showed reduced specificity (56.7%) in this stage at its optimal cutoff of 22.50 mm². Confirmed-DPN revealed near-perfect diagnostic accuracy for both *E*_mean_ (AUC = 0.979) and the combination (AUC = 0.995), with both maintaining 100% specificity. The *E*_mean_ cutoff increased to 51.79 kPa in this advanced stage.

Integrated analysis of Table 4 data revealed distinct stage-dependent patterns in diagnostic accuracy. The diagnostic value of CSA increased with disease progression, with AUC improving from 0.515 (95% CI: 0.405–0.625) in subclinical-DPN to 0.874 (95% CI: 0.811–0.937) in confirmed-DPN. In contrast, *E*_mean_ showed good diagnostic performance even in early stages (subclinical AUC = 0.730) and reached near-perfect 0.979 (95% CI: 0.956–1.000) in confirmed stage.

*E*_mean_ + CSA demonstrated most significant improvements in early stages: subclinical AUC increased from 0.730 (*E*_mean_ alone) to 0.744, with sensitivity substantially improving from 38.5% to 65.4%. In confirmed-DPN, while *E*_mean_ + CSA achieved AUC = 0.995 (95% CI: 0.987–1.000), its absolute improvement over *E*_mean_ alone (AUC = 0.979, ΔAUC = 0.016) was smaller than in early stages (ΔAUC = 0.014).

## 4. Discussion

Our study revealed significantly higher values of tibial nerve CSA and mean value of the modulus of elasticity (*E*_mean_) in the overall case group compared to the control group. These findings illustrate that NUS possesses the capability to distinguish between normal and diseased tibial nerves by detecting characteristic neuromorphological changes, after stratification and combined diagnosis, this study demonstrated higher sensitivity (92.3%) in the confirmed-DPN group compared to the single ultrasound technique diagnosis reported by Wang et al.^[23]^“Unlike Wei et al^[24]^ who reported SWE AUC = 0.836 for undifferentiated DPN, our stage-specific approach achieved superior accuracy (AUC = 0.849–0.995), highlighting the value of stratification. The diagnostic performance of SWE for DPN was found to be excellent, underscoring its substantial potential as a valuable and noninvasive adjunct in managing patients with DPN. These results align with those reported in a meta-analysis conducted by Dong.^[17]^ However, the results of our study suggest that the diagnostic sensitivity of both NUS and SWE technologies is not high (CSA: 42.3%; *E*_mean_: 54.9%).

The research^[25]^ suggests that DPN is a multiple neuropathy according to the characteristics of ultrasound images. Hence, to advance auxiliary diagnosis in quantifying and staging DPN, additional research endeavors are necessary. In this study, The diagnostic stratification criteria from the Chinese Medical Association^[20]^ were applied. In accordance with the varying diagnostic stratified subgroups, a hierarchical analysis comparing the ultrasound techniques for tibial nerve CSA and mean elasticity modulus (*E*_mean_) values was conducted. Remarkably, our study evaluated the diagnostic efficacy of NUS and SWE technologies across different stages of DPN (subclinical, suspected, clinical, and confirmed stages). We found that SWE consistently outperformed NUS at all stages, with NUS only demonstrating reliability in the late stages of the disease. This aligns with prior studies demonstrating the sensitivity of tissue stiffness measurements (*E*_mean_) to early neural degeneration.^[26]^ The progressive increase in optimal *E*_mean_ cutoffs (from 39.84 kPa in suspected-DPN to 51.79 kPa in confirmed-DPN). Mirrors the histopathological progression of nerve fibrosis observed in animal models.^[27]^ For advanced lesions, SWE alone approached optimal performance. This suggests that changes in tissue hardness occur during the early stages of DPN, even when there are no apparent morphological alterations in the affected nerves. These changes become more pronounced as the disease progresses, particularly in its advanced stages. These quantitative thresholds likely reflect underlying pathophysiological changes, such as axonal loss and collagen deposition, providing a mechanistic basis for their diagnostic utility. Our establishment of stage-specific diagnostic thresholds advances the clinical applicability of SWE, enabling more precise disease staging and monitoring.

The most striking finding of this study is the marked improvement in sensitivity (65.4%) achieved by the combined approach of NUS + SWE for subclinical-DPN, compared to either modality alone (CSA: 28.8%; *E*_mean_: 38.5%). This 26.9% increase in sensitivity addresses a critical unmet need in early DPN detection, when therapeutic interventions may halt or reverse disease progression. While previous studies^[28]^ have documented the independent value of CSA and *E*_mean_, our work is the first to systematically demonstrate their synergistic effects across the DPN spectrum. This supports the emerging paradigm of multimodal nerve assessment, where structural and functional parameters complement each other to enhance diagnostic accuracy. The combined model’s ability to maintain reasonable specificity (81.7%) while significantly improving sensitivity in early stages is particularly noteworthy. This balance is crucial for screening applications, where false positives can lead to unnecessary interventions. The higher specificity of the combined approach in suspected-DPN (93.3% vs 71.7% for SWE alone) further highlights its clinical value in differentiating true pathology from borderline cases.

Our findings challenge the conventional reliance on nerve enlargement (CSA) as a primary diagnostic marker for DPN.^[28]^ While CSA remains useful for confirming advanced DPN (specificity: 85.0%), its limited sensitivity in early stages (28.8–34.2%) necessitates complementary functional assessment. This may explain the inconsistent performance of NUS in prior DPN screening studies.^[29]^ In advanced stages, the diminishing added value of CSA suggests that SWE alone may suffice for diagnosis, simplifying clinical workflows without compromising accuracy. The reduced specificity of CSA in clinical-DPN (56.7%) is another critical observation. This decline may reflect the heterogeneity of nerve morphological changes in later disease stages, where factors such as edema or atrophy could confound CSA measurements. In contrast, *E*_mean_’s stability in advanced DPN (specificity: 100%) reinforces its reliability as a standalone marker for confirming disease severity.

Several limitations exist in our study. Firstly, the cross-sectional design precludes assessment of how diagnostic parameters evolve in individual patients over time. Additionally, the present research is confined to a solitary center, our study population was drawn from a single tertiary center, potentially limiting generalizability. Third, the optimal cutoffs identified require validation in independent cohorts with standardized acquisition protocols. Fourth, BMI differed significantly between DPN subgroups, potentially confounding nerve measurements. Future studies should adjust for anthropometric factors. Fifth, the analysis unit of this study is nerves rather than individual patients, which may introduce bias in statistical analysis. Future studies should consider intraindividual correlations or use unilateral nerve data. Sixth, blood glucose control (HbA1c) and disease duration are important risk factors for DPN, but incomplete data prevented their inclusion in the baseline analysis. Future prospective studies will systematically analyze these factors.

Future research should focus on longitudinal studies to validate the predictive value of these biomarkers for DPN progression, and investigate whether combined NUS + SWE can improve therapeutic monitoring. The development of integrated devices capable of simultaneous CSA and *E*_mean_ measurement could facilitate clinical translation of this approach.

In conclusion, this study establishes an evidence-based, stage-specific framework for DPN diagnosis. The combined NUS + SWE approach offers unparalleled advantages for early detection, while SWE alone may suffice in advanced stages. These findings advocate for updating diagnostic algorithms to integrate structural and functional nerve assessments, paving the way for earlier interventions and improved patient outcomes. The development of standardized protocols and accessible technologies will be critical to translate these advances into routine clinical practice.

## Acknowledgments

We gratefully acknowledge the contributions and efforts of all patients who participated in this study.

## Author contributions

**Data curation:** Bo-yu She.

**Formal analysis:** Meng-lu Song.

**Investigation:** Zhen-han Lai, Hua-qiang Zheng.

**Methodology:** Jia-ying Wang, Kun-bin Wu.

**Project administration:** Guo-rong Lyu.

**Writing – original draft:** Zhen-han Lai.

**Writing – review & editing:** Wen-ting Jiang.
